# Exploratory Study on the Association Between Pre-treatment Factors and Hematological Toxicity in Colorectal Cancer Patients Receiving CapeOX (Capecitabine Plus Oxaliplatin) Therapy

**DOI:** 10.7759/cureus.118004

**Published:** 2026-10-16

**Authors:** Makoto Kishimoto, Jin Tokunaga

**Affiliations:** 1 First Department of Clinical Pharmacy, Faculty of Pharmaceutical Sciences, Kyushu University of Medical Science, Nobeoka, JPN; 2 Department of Pharmacy, Kirishima Medical Center, Kirishima, JPN; 3 Faculty of Pharmaceutical Sciences, Kyushu University of Medical Science, Nobeoka, JPN

**Keywords:** adverse effect, capeox therapy, colorectal cancer, cut-off value, hematologic toxicity, inflammatory marker, predictor

## Abstract

Background: The capecitabine plus oxaliplatin (CapeOX) regimen is commonly used for adjuvant treatment and selected first‑line treatment settings in colorectal cancer. However, hematologic toxicities, such as leukopenia, neutropenia, anemia, and thrombocytopenia, may adversely affect treatment continuity, resulting in treatment delays, dose reductions, or treatment discontinuation. If these toxicities could be predicted based on pre-treatment blood test results and markers of inflammation and nutrition, it would lead to improved treatment continuity. In this study, we explored potential associations related to hematologic toxicities that occur at the end of the first cycle of CapeOX therapy.

Methods: A retrospective analysis was conducted on 108 patients with colorectal cancer who began CapeOX therapy between September 2020 and October 2023. We evaluated hematologic toxicity (Grade ≥ 1) at the end of the first treatment cycle based on the Common Terminology Criteria for Adverse Events (CTCAE) version 5.0 and examined its association with pre-treatment blood test results, nutritional indices (PNIs), and inflammatory markers (systemic inflammation response index (SIRI), systemic immune‑inflammation index (SII), neutrophil‑to‑lymphocyte ratio (NLR), platelet‑to‑lymphocyte ratio (PLR), and lymphocyte‑to‑monocyte ratio (LMR)). We performed multivariate logistic regression analysis on factors showing significant differences and calculated cutoff values using receiver operating characteristic (ROC) analysis.

Results: An SII value of ≤496.8 was identified as a significant pretreatment factor associated with the development of leukopenia. For hemoglobin reduction, age ≥68 years, red blood cell count ≤394 × 10⁴/μL, and hemoglobin (Hb) ≤11.7 g/dL were associated; for thrombocytopenia, lymphocyte count ≤12.41 × 10²/μL and SII ≤477.9 were significantly associated. The identified associations may help generate hypotheses for future prospective studies of pre‑treatment risk assessment and supportive‑care strategies.

Conclusions: Evidence suggests that some of the hematologic toxicities associated with CapeOX therapy show exploratory associations with pre-treatment blood tests and markers of inflammation and nutrition. These exploratory associations may help inform future hypothesis‑generating research on pretreatment risk assessment and supportive‑care strategies.

## Introduction

Colorectal cancer is one of the leading malignant tumors in Japan, with both incidence and mortality rates remaining high, partly because a substantial proportion of cases present with symptoms related to metastatic disease [1,2]. The capecitabine plus oxaliplatin (CapeOX) regimen is commonly used for adjuvant treatment and selected first‑line treatment settings in colorectal cancer [3]. However, it has been reported that CapeOX therapy is associated with a variety of adverse effects, including peripheral neuropathy, hand-foot syndrome, gastrointestinal symptoms, and hematologic toxicity, which can lead to treatment delays, dose reductions, or discontinuation [4,5].

In recent years, efforts have been made to predict the occurrence of adverse effects using pre-treatment clinical data. For example, with regard to the prediction of early adverse effects in CapeOX therapy, factors such as age, body build, renal function, and muscle mass have been shown to be associated with these effects, and models using machine learning have achieved a certain level of predictive accuracy [4].

Furthermore, it has been suggested that pre-treatment blood test results and markers of inflammation and nutritional status may serve as predictive factors for non-hematologic adverse effects associated with CapeOX therapy, such as peripheral neuropathy, nausea, loss of appetite, and diarrhea [5].

However, there are few studies that have systematically examined have systematically examined predictive factors, focusing on hematologic toxicity (leukopenia, neutropenia, anemia, and thrombocytopenia), using pre-treatment blood test results and markers of inflammation and nutrition. Although several studies have examined pretreatment laboratory markers as predictors of non‑hematologic adverse effects during CapeOX therapy, published research specifically evaluating hematologic toxicities using pretreatment blood tests is extremely limited. For example, Kishimoto et al. [5] investigated pretreatment predictors of peripheral neuropathy, nausea, anorexia, and diarrhea, but did not evaluate hematologic toxicity. Hematologic toxicity is a significant adverse effect that directly impacts treatment continuity; if its occurrence can be predicted in advance, treatment discontinuation can be avoided through enhanced supportive care and dose adjustments, thereby improving safety.

Therefore, this study aimed to explore potential associations between pre‑treatment blood test results, inflammatory markers, and the development of hematologic toxicity in colorectal cancer patients undergoing CapeOX therapy. The primary objective was to examine associations between prespecified pre‑treatment laboratory variables and grade ≥1 hematologic toxicity during the first CapeOX cycle. The secondary objectives were exploratory assessment of discriminative performance and internally derived candidate thresholds, which will require validation in independent cohorts.

## Materials and methods

This study is a retrospective secondary analysis using existing data on colorectal cancer patients who received CapeOX therapy, and its objective was to exploratively identify pre‑treatment factors associated with hematologic toxicity [5]. Although the dataset was constructed in a previous study, this study extracted only variables related to hematologic toxicity and constructed an independent analytical model. The dataset was originally developed in a previously published analysis of non‑hematologic adverse effects associated with CapeOX therapy (Kishimoto M, Koinuma M, Tokunaga J: Exploratory investigation of predictive factors for reducing adverse effects in CapeOX therapy for colorectal cancer patients. Cureus. 2025, 17:95702 [5]). The present study represents an independent secondary analysis focusing specifically on hematologic toxicity. Neither the original study nor this secondary analysis was part of a registered clinical trial. In accordance with the institutional opt‑out policy for retrospective studies with waived informed consent, patients who explicitly requested exclusion from research use of their clinical data were not included in the dataset. No patients discontinued CapeOX therapy before completing the first cycle; therefore, no patients were excluded for this reason. Although treatment discontinuation before completing the first cycle could theoretically introduce selection bias, because such discontinuation may be related to toxicity or clinical deterioration, no patients met this criterion in the present dataset. Therefore, the impact of this potential bias is considered minimal.

Patients with prior radiotherapy and previous chemotherapy, including adjuvant therapy, liver metastases, bone‑marrow involvement, or baseline cytopenias (leukopenia, neutropenia, anemia, or thrombocytopenia before treatment initiation), were not included in this study. All patients were chemotherapy‑naïve and received capecitabine plus oxaliplatin (CapeOX) as first‑line treatment.

The analysis included 108 colorectal cancer patients who began CapeOX therapy at the Kirishima City Medical Association Medical Center between September 1, 2020, and October 31, 2023. All patients were chemotherapy-naïve at the start of treatment; the analysis included cases for which pre-treatment blood test results and adverse event assessments at the end of the first treatment cycle were available. Treatment intent was also documented. Among the 108 patients, 98 received CapeOX as adjuvant chemotherapy for Stage II-III colorectal cancer, while 10 patients with Stage IV disease received CapeOX as first‑line therapy for metastatic colorectal cancer. All patients were chemotherapy‑naïve at treatment initiation. Patients who refused to participate in the study, those who discontinued treatment on their own initiative, and cases with missing blood test results or adverse event information were excluded. A total of 108 patients were screened, and no cases were excluded. All patients had complete pretreatment laboratory data and cycle 1 adverse‑event assessments; therefore, the final analysis included all 108 screened patients. One case involved a synchronous lesion located in the jejunum. Although the jejunum is not a colorectal site, chemotherapy was administered according to colorectal cancer treatment protocols; therefore, the case was retained in the colorectal cancer cohort for analysis.

This study did not involve any new interventions or additional data collection. Data collection was conducted within the scope of the ethical approval obtained for the previous study, and personal information was anonymized and managed accordingly. Ethical approval was obtained from the Ethics Review Committee of the Kirishima City Medical Association Medical Center (Receipt No.: 202301) and the Ethics Review Committee of Kyushu University of Health and Welfare (Approval No.: 23-015). Since this was a retrospective study, written informed consent was waived, and an opt-out procedure via the institution’s website was adopted. In accordance with the institutional opt‑out policy for retrospective studies with waived informed consent, patients who explicitly requested exclusion from research use of their clinical data were not included in the dataset. No patients discontinued CapeOX therapy before completing the first cycle; therefore, no patients were excluded for this reason.

The CapeOX regimen was administered in 21-day cycles, consisting of an intravenous infusion of 130 mg/m² of oxaliplatin on Day 1, followed by oral administration of 1,000 mg/m² of capecitabine twice daily from Day 1 through Day 14. Dose adjustments were made at the discretion of the treating physician. Dose (%) was defined as the percentage of the planned chemotherapy dose that was actually administered during the first cycle (relative dose intensity). No dose reductions or treatment delays occurred during Cycle 1. All patients received the planned CapeOX doses during the first cycle, and hematologic toxicity was assessed at the standard Day 21 evaluation.

To identify predictors of hematologic toxicity, we extracted pre‑treatment hematologic parameters (white blood cell count, neutrophil count, lymphocyte count, monocyte count, red blood cell count, hemoglobin, and platelet count; all measured in ×10³/µL, except hemoglobin in g/dL) and biochemical parameters (albumin in g/dL, C‑reactive protein (CRP) in mg/dL, aspartate aminotransferase (AST), and alanine aminotransferase (ALT) in U/L, serum creatinine in mg/dL, estimated glomerular filtration rate (eGFR) in mL/min/1.73 m², and creatinine clearance in mL/min). Furthermore, we calculated the following inflammatory and nutritional indices: prognostic nutritional index (PNI) (10 × albumin + 0.005 × lymphocytes), systemic inflammation response index (SIRI) (neutrophils × monocytes / lymphocytes), systemic immune‑inflammation index (SII) (platelets × neutrophils / lymphocytes), neutrophil‑to‑lymphocyte ratio (NLR) (neutrophils / lymphocytes), platelet‑to‑lymphocyte ratio (PLR) (platelets / lymphocytes), and lymphocyte‑to‑monocyte ratio (LMR) (lymphocytes / monocytes). Pre-treatment laboratory values were obtained on the day of Cycle 1 initiation (Day 1). Reference ranges for all laboratory, nutritional, and inflammatory indices used in this study are provided in Appendix A.

Smoking history was categorized using the Brinkman index as recorded in the electronic medical record. Drinking history categories were taken directly from the electronic medical record, where alcohol consumption is routinely documented using predefined clinical categories. These categories were not based on a numerical index.

Hematologic toxicity (leukopenia, neutropenia, anemia, and thrombocytopenia) was classified as Grade 0 or Grade ≥1 based on the Common Terminology Criteria for Adverse Events (CTCAE) version 5.0 (Japanese version) [6] using routine blood tests conducted at the end of each cycle. Post‑treatment blood tests used to assess hematologic toxicity at the end of the first cycle were performed on Day 21, which is the standard timing for cycle‑end laboratory evaluation in our institution. All hematologic parameters were Grade 0 at baseline, and patients with Grade ≥1 leukopenia, neutropenia, anemia, or thrombocytopenia before starting CapeOX were not included in the corresponding toxicity analyses. Baseline CTCAE grades were confirmed and reported to clarify that the outcomes represent abnormalities observed at the end of cycle 1. No supportive interventions such as G‑CSF administration, blood transfusion, or other hematologic supportive measures were given during Cycle 1 before the end‑of‑cycle blood test. Therefore, hematologic toxicity grading reflects untreated laboratory values.

In the statistical analysis, we first evaluated the association between pre-treatment factors and each toxicity using univariate analysis. For continuous variables, we used the Wilcoxon two-sample test and reported the Z-statistic and p-value. For categorical variables, we used Pearson’s χ² test or Fisher’s exact test and reported the χ² statistic or exact probability and p-value. Factors that showed significant differences in the univariate analysis were included in multivariate logistic regression analysis, and the odds ratio (OR) and 95% confidence interval (CI) were reported. In addition, ROC analysis was performed to evaluate predictors of toxicity occurrence, and the AUC and cutoff values were calculated. Fisher’s exact test was used to compare groups based on cutoff values. The significance level was set at p < 0.05. Cutoff values for each variable were determined using ROC curve analysis within the same dataset. Because no internal validation (e.g., bootstrapping or cross‑validation) was performed, these cutoff values should be interpreted as exploratory and data‑dependent rather than as established thresholds for clinical risk stratification.

In the multivariate logistic regression analysis, Grade 0 was coded as 0 and Grade ≥1 as 1. Therefore, the ORs indicate the likelihood of developing hematologic toxicity (Grade ≥1).

Some ORs in the multivariable analysis, particularly for basophils, SIRI, and CRP, showed extremely wide confidence intervals due to sparse data and separation. These values were retained for transparency, but their instability was acknowledged, and interpretation was limited accordingly.

We performed ROC analysis on the independent variables to calculate the cutoff value, AUC, sensitivity, and specificity. We stratified patients into two groups based on the cutoff value and compared the incidence rates of toxicity. Statistical analysis was performed using JMP® Student Edition version 19.0.5 (SAS Institute Inc., 2025, jmp.com). To maintain consistency with our previous report, continuous variables were summarized using mean ± SD. The Wilcoxon rank‑sum test was applied to reduce the influence of potential outliers rather than to address non‑normality. Multiple univariate comparisons were performed across numerous variables and four hematologic toxicities. These analyses were considered exploratory, and no formal correction for multiple testing was applied. Because JMP does not provide confidence intervals for the area under the curve (AUC), the standard error (SE) of the AUC was estimated according to the method of Hanley and McNeil [7]. The 95% confidence interval (CI) for the AUC was calculated as AUC ± 1.96 × SE. Sensitivity and specificity were obtained from the ROC output in JMP at the cutoff value that maximized the Youden index (sensitivity − (1 − specificity)). In addition, several continuous predictors showed unstable ORs due to sparse data and separation. Therefore, we retained the original numerical estimates for transparency but simplified the multivariable model by reducing the number of predictors. The ORs for continuous variables were interpreted on their original scales; for example, the age OR represents a per‑one‑year increment.

## Results

Previous studies have suggested that pre-treatment blood test results and markers of inflammation and nutrition may be associated with nonhematologic adverse events of CapeOX therapy. In this study, using the same dataset, we focused on hematologic toxicity and analyzed the association between the occurrence of leukopenia, neutropenia, anemia, and thrombocytopenia and pre-treatment factors.

Leukopenia

Table 1 and Table 2 show the results of a comparison of pre-treatment factors associated with leukopenia. The results of the multivariate logistic regression analysis are shown in Table 3A. In cases of leukopenia, the SII at the start of the first cycle was significantly lower in the Grade ≥1 group (334.52 ± 92.71) compared to the Grade 0 group (630.69 ± 385.02) (Z = -3.2606, p = 0.0022) (Table 2). Multivariate logistic regression analysis identified SII as the sole independent factor associated with the occurrence of leukopenia (OR = 0.9660, p = 0.0098) (Table 3A). Lower baseline SII was associated with first‑cycle leukopenia in the exploratory multivariable analysis; the internally derived threshold of 496.768 requires validation in an independent cohort. In the ROC analysis, this threshold demonstrated good discriminatory ability with an AUC of 0.8013 (Table 4A). Lower baseline SII was associated with first‑cycle leukopenia in this cohort; however, this in‑sample association does not establish clinical utility, and its potential role in pre‑treatment risk assessment requires external validation. Based on the above, SII was identified as a pre‑treatment factor associated with an increased risk of leukopenia. Additional baseline comparisons across all four hematologic toxicities are summarized in Appendix B.

**Table 1:** Pre‑treatment factors associated with leukopenia (Grade ≥1) Values marked with an asterisk (*) are statistically significant (p < 0.05). Statistical tests used: a) Wilcoxon two-sample test; b) Pearson's chi-square test.

Variable		Grade 0	Grade ≥ 1	Statistic (Z / χ²)	p-value	
		n = 97	n = 11			
Age (years)		66.68 ± 10.40	68.27 ± 8.58	Z = 0.3863	0.6992	a)
Sex	Male	55	4	χ² = 1.7112	0.1991	b)
	Female	42	7			
BMI (kg/m²)		22.10 ± 3.72	22.64 ± 2.99	Z = 0.4571	0.6476	a)
Body surface area (m²)		1.57 ± 0.20	1.55 ± 0.14	Z = -0.5180	0.6044	a)
General condition（PS）	0	85	10	χ² = 1.1357	0.5965	b)
	1	8	1			
Smoking history	0	38	5	χ² = 0.0032	0.5965	b)
	1-199	9	1			
	200-399	6	2			
	400-699	13	1			
	700-	31	2			
Drinking history	None	30	2	χ² = 5.8957	0.1912	b)
	Occasional	21	0			
	Weekly	17	1			
	Everyday	29	6			
Cancer site	Upper colon	12	0	χ² = 8.1922	0.2790	b)
	Lower colon	5	0			
	Sigmoid colon	21	3			
	Transverse colon	19	0			
	Jejunum	1	0			
	Rectum	32	4			
	Cecum	8	3			
Stage	Ⅱa	23	1	χ² = 4.3770	0.9702	b)
	Ⅱb	5	1			
	Ⅲa	13	1			
	Ⅲb	36	7			
	Ⅲc	10	1			
	Ⅳ	10	0			
Dose(%)	Oxaliplatin	89.25 ± 7.74	87.36 ± 9.54	Z = -0.6094	0.5422	a)
	Capecitabine	92.90 ± 9.82	94.97 ± 11.59	Z = 1.0462	0.2954	a)

**Table 2:** Comparison of blood test values, nutritional and inflammatory indices before treatment by adverse effect after one cycle Values marked with an asterisk (*) are statistically significant (p < 0.05). Statistical tests used: Wilcoxon two-sample test. CRP: C-reactive protein; AST: aspartate aminotransferase; ALT: alanine aminotransferase; Hb: hemoglobin; eGFR: estimated glomerular filtration rate; Cr: creatinine; Ccr: creatinine clearance; PNI: prognostic nutrition index; SIRI: systemic inflammatory response index; NLR: neutrophil-to-lymphocyte ratio; PLR: platelet-to-lymphocyte ratio; LMR: lymphocyte-to-monocyte ratio; SII: systemic immune inflammatory index

Variable	Reference range (normal value)	Leukopenia				Neutropenia				Hemoglobin decrease				Thrombocytopenia			
		Grade 0	Grade ≥ 1	Statistic (Z)	p-value	Grade 0	Grade ≥ 1	Statistic (Z)	p-value	Grade 0	Grade ≥ 1	Statistic (Z)	p-value	Grade 0	Grade ≥ 1	Statistic (Z)	p-value
		n = 97	n = 11			n = 93	n = 15			n = 71	n = 37			n = 82	n = 26		
Red blood cell (10^6^/μL)	Male 420–570 / Female 380–500	422.60±65.98	379.09±44.13	-2.7986	0.0051*	422.06±64.29	394.06±68.55	-1.8124	0.0699	439.81±63.08	376.64±47.31	-5.9335	<0.0001*	424.35±67.65	398.69±53.82	-2.2931	0.0209*
																		White blood cell (10^3^/μL)	35–90	62.27±41.07	35.81±7.41	-4.6285	<0.0001*	62.44±42.02	41.86±10.23	-3.8880	0.0001*	62.23±47.07	54.48±18.88	-0.8710	0.3837	62.82±44.42	49.34±15.50	-2.0767	0.0218*
Basophil (10^3^/μL)	0-2	0.32±0.27	0.17±0.09	-1.9607	0.0499*	0.32±0.27	0.22±0.19	-1.6970	0.0888	0.32±0.29	0.28±0.17	-0.1521	0.8791	0.33±0.28	0.24±0.16	-1.3871	0.1654
																		Eosinophil (103/μL)	0-6	2.23±2.21	1.29±0.74	-1.9249	0.0542	2.22±2.26	1.63±0.88	-0.7640	0.4449	2.42±2.45	1.58±1.15	-2.8549	0.0043*	2.13±2.30	2.15±1.48	0.5317	0.5949
Neutrophil (10^3^/μL)	40-75	38.45±28.88	20.32±4.32	-4.4541	<0.0001*	38.95±29.35	22.09±6.28	-4.4995	<0.0001*	37.54±32.33	34.81±16.78	-0.5535	0.5799	38.89±31.11	29.41±11.53	-2.2599	0.0238*																		
																		Lymphocyte (10^3^/μL)	20-50	17.30±10.20	11.44±3.12	-2.9051	0.0037*	16.98±10.41	14.94±5.34	-0.6040	0.5458	17.97±11.46	14.27±5.01	-2.0618	0.0392*	17.66±10.94	13.68±4.12	-2.0767	0.0378*
Monocyte (10^3^/μL)	2-10	3.95±2.23	2.57±0.60	-3.1794	0.0015*	3.95±2.27	2.95±0.93	-2.3764	0.0175*	3.96±2.48	3.52±1.33	-0.6020	0.5471	3.80±2.28	3.85±1.79	0.0035	0.9971																		
																		Platelet (10^4^/μL)	15-35	25.91±7.68	18.47±5.01	-3.4792	0.0005*	25.50±7.74	23.02±7.83	-1.3281	0.1841	24.08±6.75	27.22±9.16	1.6573	0.0974	26.70±6.95	20.28±8.31	-4.8688	<0.0001*
Serum Albumin (g/dL)	3.8-5.0	4.04±0.47	4.07±0.24	-0.3007	0.7636	4.05±0.44	4.04±0.54	0.0490	0.9609	4.17±0.37	3.81±0.51	-3.4171	0.0006*	4.07±0.44	3.97±0.49	-0.7535	0.4511																		
																		CRP (mg/dL)	<0.3	0.38±1.15	0.06±0.07	-2.2663	0.0234*	0.38±1.17	0.14±0.31	-2.2811	0.0225*	0.22±0.38	0.60±1.78	0.7872	0.4311	0.39±1.24	0.22±0.30	1.0724	0.2835
AST (IU/L)	13-33	21.30±11.68	18.63±5.86	-0.7277	0.4668	21.00±11.48	21.26±10.01	0.0667	0.9468	19.38±7.34	24.21±15.97	1.1221	0.2618	21.43±12.38	19.76±6.46	0.0360	0.9713																		
																		ALT (IU/L)	8-42	20.55±11.88	16.54±8.69	-1.2916	0.1965	19.52±10.86	24.00±15.48	0.7960	0.4260	19.70±9.93	21.00±14.46	-0.2463	0.8054	20.25±11.98	19.80±10.67	0.1007	0.9198
Hb (g/dL)	Male 13.0–17.0 / Female 11.5–15.0	12.80±3.31	11.32±1.12	-2.2151	0.0267*	12.78±3.34	11.84±1.91	-1.3241	0.1855	13.67±3.46	10.70±0.97	-7.6869	<0.0001*	12.87±3.54	11.96±1.51	-1.5347	0.1248																		
																		eGFR (mL/min/1.73m^2^)	≥60	78.03±20.71	72.36±12.73	-1.0923	0.2747	76.86±20.62	81.13±16.40	0.8930	0.3718	79.07±21.77	74.35±16.15	-1.4343	0.1515	78.18±20.79	75.15±17.78	-1.0782	0.2809
Cr (mg/dL)	Male 0.65–1.07 / Female 0.46–0.79	0.70±0.17	0.70±0.15	-0.0609	0.9514	0.71±0.17	0.67±0.12	-0.7020	0.4826	0.71±0.17	0.69±0.17	-1.1138	0.2653	0.69±0.16	0.74±0.17	0.9812	0.3265																		
																		Ccr (mL/min)	80-130	162.49±44.09	159.89±33.49	0.1218	0.903	160.57±44.05	172.49±35.36	1.4835	0.1379	170.49±43.74	146.37±37.18	-2.9196	0.0035*	163.73±42.33	157.46±45.58	-0.4563	0.6482
PNI	≥45(Good)	49.12±7.00	46.45±2.70	-1.6861	0.0918	49.01±6.93	47.87±5.44	-0.3553	0.7223	50.68±6.71	45.32±5.27	-4.3502	<0.0001*	49.57±7.04	46.57±5.13	-2.1809	0.0292*																		
SIRI	1.0-1.5(clinical cut-off)	0.98±0.82	0.47±0.15	-3.0676	0.0022*	1.00±0.82	0.46±0.21	-3.8732	0.0001*	0.90±0.82	0.97±0.73	0.3884	0.6977	0.92±0.79	0.95±0.82	-0.0610	0.9513
NLR	1.0-3.0	2.40±1.10	1.86±0.52	-1.4830	0.1381	2.47±1.09	1.59±0.48	-3.1714	0.0015*	2.21±0.93	2.61±1.27	1.3918	0.164	2.38±1.11	2.26±0.93	-0.1760	0.8603
PLR	100-200	1.73±076	1.65±0.37	0.0914	0.9272	1.74±0.76	1.60±0.49	-0.3820	0.7025	1.55±0.63	2.04±0.80	3.2562	0.0011*	1.77±0.77	1.56±0.57	-1.1317	0.2577
LMR	3.0-5.0	4.79±2.08	4.49±0.97	-0.0914	0.9272	4.70±2.08	5.14±1.30	1.5368	0.1243	4.89±1.94	4.52±2.10	-1.2558	0.2092	4.98±2.06	4.08±1.64	-2.0228	0.0431*
SII	≤500(Increased by inflammation and tumors)	630.69±385.02	334.52±92.71	-3.2606	0.0022*	638.85±387.94	362.94±159.15	-3.1892	0.0014*	525.47±257.28	744.56±510.51	1.7673	0.0772	651.68±404.45	439.20±204.17	-2.6120	0.0090*

**Table 3:** Multivariable logistic regression analysis of significant factors Values marked with an asterisk (*) are statistically significant (p < 0.05). Statistical tests used: multivariable logistic regression analysis. Sex was treated as a categorical variable, with “female” as the reference category. Odds ratios (ORs) were calculated relative to females. Smoking status was treated as a categorical variable, with “non-smoker” as the reference category. Odds ratios (ORs) were calculated relative to non-smokers. Drinking status was treated as a categorical variable, with "non-drinker" as the reference category. Odds ratios (ORs) were calculated relative to non-drinkers. CRP: C-reactive protein; Hb: hemoglobin; Ccr: creatinine clearance; PNI: prognostic nutrition index; SIRI: systemic inflammatory response index; NLR: neutrophil-to-lymphocyte ratio; PLR: platelet-to-lymphocyte ratio; LMR: lymphocyte-to-monocyte ratio; SII: systemic immune inflammatory index

		Odds ratio	95%CI	p-value
(A) Leukopenia				
Red blood cell (10^6^/μL)		1.0063	0.9531-1.0728	0.8454
					White blood cell (10^3^/μL)		0.7853	0.1656-2.8359	0.7154
Basophil (10^3^/μL)		1.0275×10^-4^	0.03693-3.82×10^12^	0.1623					
					Neutrophil (10^3^/μL)		1.8830	0.3430-14.2900	0.4675
Lymphocyte (10^3^/μL)		0.2138	0.0182-1.2930	0.0961					
					Monocyte (10^3^/μL)		11.6200	0.0222-1.0000×10^4^	0.4591
Platelet (10^4^/μL)		1.7640	0.9283-4.2670	0.0854					
					CRP (mg/dL)		1.3310×10^-8^	3.1400×10^-21^-1.3950	0.0583
Hb (g/dL)		0.6370	0.10850-1.4940	0.5940					
					SIRI		2.9000×10^-4^	6.29×10^-20^-37490×10^4^	0.5790
SII		0.9660	0.9180-0.9930	0.0098*					
					(B) Neutropenia				
BMI (kg/m²)		1.1507	0.9614-1.4080	0.1285
					White blood cell (10^3^/μL)		0.8850	0.6080-1.2020	0.4537
Neutrophil (10^3^/μL)		1.0264	0.6140-1.8860	0.9249					
					Monocyte (10^3^/μL)		1.4680	0.2200-17.0200	0.7207
CRP (mg/dL)		0.6840	0.02050-7.6500	0.8000					
					SIRI		0.1705	7.03×10^-7^-2.941×10^2^	0.7175
NLR		0.4120	0.0080-9.4200	0.5959					
					SII		0.9996	0.9930‐1.0055	0.9071
(C) Hemoglobin decrease									
Age (years)		1.3410	1.0300-2.8500	0.0229*
					Sex		6.2300	0.3860-151.5000	0.0605
BMI (kg/m²)		0.7950	0.2790-1.4510	0.457					
					Body surface area (m²)		0.5560	0.0225-10.6700	0.1933
Smoking history	1-199	0.5540	0.0280-10.9300	0.6984					
	200-399	1.4380	0.0737-2.8090	0.8105
	400-699	0.1930	0.0089-4.1800	0.2943
	700-	0.1880	0.0085-4.1300	0.289
Drinking history	Occasional	0.5540	0.0280-10.9300	0.6984
	Weekly	1.0360	0.0737-28.0900	0.8105
	Everyday	0.1930	0.0089-4.1800	0.2943
Red blood cell (10^6^/μL)		0.9420	0.9370-0.9997	0.0420*
					Eosinophil (103/μL)		0.6410	0.2530-1.3450	0.2710
Lymphocyte (10^3^/μL)		0.9390	0.7650-1.1400	0.5230					
					Serum albumin (g/dL)		1.4180	0.1460-10.9900	0.7419
Hb (g/dL)		0.0623	0.0098-0.2198	0.0003*					
					Ccr		0.9831	0.9460-1.0168	0.3491
PNI		1.1180	0.8680-1.4490	0.3730					
					PLR		0.6490	0.3530-1.2440	0.1649
(D) Thrombocytopenia									
Capecitabine dose (%)		1.0496	0.9880-1.1197	0.1206
					Red blood cell (10^6^/μL)		0.9914	0.9782-1.0041	0.1856
White blood cell (103/μL)		1.4480	0.9140-2.3730	0.1155					
					Neutrophil (10^3^/μL)		0.7470	0.4400-1.2580	0.2708
Lymphocyte (10^3^/μL)		0.4810	0.2250-0.9430	0.0326*					
					Platelet (10^4^/μL)		1.0218	0.8167-1.2530	0.8406
PNI		0.8780	0.7320-1.0400	0.1351					
					LMR		0.9310	0.4090-1.9370	0.8553
SII		0.9910	0.9820-0.9996	0.0382*					

**Table 4:** Significant predictors and cutoff values by adverse effect and comparisons before and after cutoff Values marked with an asterisk (*) are statistically significant (p < 0.05). Statistical tests used: Fisher's exact test. Hb: hemoglobin; SII: systemic immune inflammatory index

	Cut-off value	AUC	AUC 95%CI	Sensitivity	Specificity		Grade0	Grade ≥ 1	p-value
(A) Leukopenia									
SII	496.768	0.8013	0.628 - 0.975	0.9091	0.5361	≦496.768	40 (80.00%)	10 (20.00%)	0.0025*
						>496.768	57 (98.28%)	1 (1.72%)	
(B) Hemoglobin decrease									
Age (years)	68.0	0.6917	0.561 - 0.822	0.8378	0.5493	<68.00	39 (86.67%)	6 (13.33%)	0.0001*
						≧68.00	32 (50.79%)	31 (49.21%)	
Red blood cell (10^6^/μL)	394.0	0.8491	0.714 - 0.984	0.6757	0.8873	≦394.0	8 (24.24%)	25 (75.76%)	0.0001*
						>394.0	63 (84.00%)	12 (16.00%)	
Hb (g/dL)	11.70	0.9520	0.894 - 1.000	0.9459	0.8592	≦11.7	10 (22.22%)	35 (77.78%)	0.0001*
						>11.7	61 (96.83%)	2 (3.17%)	
(C) Thrombocytopenia									
Lymphocyte (10^3^/μL)	12.41	0.6358	0.446 - 0.826	0.5385	0.7199	≦12.4100	23 (62.16%)	14 (37.84%)	0.0194*
						>12.4100	59 (83.10%)	12 (16.90%)	
SII	477.901	0.6707	0.516 - 0.825	0.7692	0.6098	≦477.901	32 (61.54%)	20 (38.46%)	0.0013*
						>477.901	50 (89.29%)	6 (10.71%)	

Neutropenia

The results of the univariate analysis of neutropenia are shown in Table 2 and Table 5, and the results of the multivariate logistic regression analysis are shown in Table 3B.

**Table 5:** Pre‑treatment factors associated with neutropenia (grade ≥1) Values marked with an asterisk (*) are statistically significant (p < 0.05). Statistical tests used: a) Wilcoxon two-sample test; b) Pearson's chi-square test.

Variable		Grade 0	Grade ≥ 1	Statistic (Z / χ²)	p-value	
		n = 93	n = 15			
Age (years)		67.46 ± 10.21	63.00 ± 9.65	Z = -1.8007	0.0717	a)
Sex	Male	52	7	χ² = 0.1500	0.5044	b)
	Female	41	8			
BMI (kg/m²)		22.05 ± 3.72	23.41 ± 4.3	Z = 1.9632	0.0496*	a)
Body surface area (m²)		1.57 ± 0.19	1.59 ± 0.21	Z = 0.7639	0.4449	a)
General condition (PS）	0	85	12	χ² = 3.5447	0.1756	b)
	1	8	3			
Smoking history	0	38	5	χ² = 5.0055	0.2366	b)
	1-199	8	2			
	200-399	6	2			
	400-699	10	4			
	700-	31	2			
Drinking history	None	31	3	χ² = 1.9445	0.5847	b)
	Occasional	18	3			
	Weekly	16	2			
	Everyday	28	7			
Cancer site	Upper colon	12	0	χ² = 7.7881	0.2540	b)
	Lower colon	5	1			
	Sigmoid colon	23	1			
	Transverse colon	17	2			
	Jejunum	1	0			
	Rectum	28	8			
	Cecum	8	3			
Stage	Ⅱa	21	3	χ² = 10.1572	0.2946	b)
	Ⅱb	3	3			
	Ⅲa	12	2			
	Ⅲb	36	7			
	Ⅲc	11	0			
	Ⅳ	10	0			
Dose (%)	Oxaliplatin	89.10 ± 7.98	89.09 ± 7.75	Z = -0.0355	0.9717	a)
	Capecitabine	92.8 ± 9.47	95.23 ± 12.86	Z = 0.9239	0.3555	a)

In patients with neutropenia, SIRI, NLR, and SII at the start of the first cycle were significantly lower in the Grade ≥ 1 group than in the Grade 0 group in univariate analysis (SIRI: Z = -3.8732, p = 0.0001; NLR: Z = -3.1714, p = 0.0015; SII: Z = −3.1892, p = 0.0014) (Table 2). However, in multivariate logistic regression analysis, none of the factors, including SIRI and NLR, showed a significant association as independent predictors (p > 0.05) (Table 3B). Based on the above, no independent predictors that increase the risk of neutropenia were identified.

Hemoglobin decrease

Pre-treatment factors associated with decreased hemoglobin are shown in Table 2 and Table 6, and the results of the multivariate logistic regression analysis are shown in Table 3C. In cases of decreased hemoglobin, the age at the start of the first cycle was significantly higher in the Grade ≥1 group than in the Grade 0 group, while the red blood cell count (×10⁴/μL) and Hb (g/dL) were significantly lower (age: Z = 3.2595, p = 0.0011; red blood cell count: Z = -5.9335, p < 0.0001; Hb: Z = -7.6869, p < 0.0001) (Tables 2, 6). The results of the multivariate logistic regression analysis identified age (OR = 1.3410, p = 0.0229), red blood cell count (OR = 0.9420, p = 0.0420), and Hb (OR = 0.0623, p = 0.0003) as independent factors associated with the occurrence of decreased hemoglobin (Table 3C). In the ROC analysis, cutoff values of age ≥68 years, red blood cell count ≤394 × 10⁴/μL, and Hb ≤ 11.7 g/dL were identified; the AUC values were 0.69, 0.78, and 0.95, respectively, with Hb demonstrating particularly high discriminatory power (Table 4B). Based on the above, age, red blood cell count, and Hb were identified as pre‑treatment factors associated with an increased risk of decreased hemoglobin levels.

**Table 6:** Pre‑treatment factors associated with hemoglobin decrease (Grade ≥1) Values marked with an asterisk (*) are statistically significant (p < 0.05). Statistical tests used: a) Wilcoxon two-sample test; b) Pearson's chi-square test.

Variable		Grade 0	Grade ≥ 1	Statistic (Z / χ²)	p-value	
		n = 71	n = 37			
Age (years)		64.54 ± 10.81	71.24 ± 7.25	Z = 3.2595	0.0011*	a)
Sex	Male	46	13	χ² = 7.5776	0.0033*	b)
	Female	25	24			
BMI (kg/m²)		23.24 ± 3.75	20.31 ± 3.17	Z = -3.9748	<0.0001*	a)
Body surface area (m²)		1.62 ± 0.18	1.47 ± 0.17	Z = -4.0201	<0.0001*	a)
General condition（PS）	0	66	31	χ² = 22.8371	0.0663	b)
	1	5	6			
Smoking history	0	23	20	χ² = 25.4196	0.0463*	b)
	1-199	1	4			
	200-399	8	0			
	400-699	12	2			
	700-	22	11			
Drinking history	None	16	18	χ² = 8.6613	0.0330*	b)
	Occasional	15	6			
	Weekly	12	6			
	Everyday	28	7			
Cancer site	Upper colon	4	8	χ² = 9.4878	0.2133	b)
	Lower colon	4	2			
	Sigmoid colon	19	5			
	Transverse colon	12	7			
	Jejunum	0	1			
	Rectum	25	9			
	Cecum	7	4			
Stage	Ⅱa	19	5	χ² = 6.5384	0.1722	b)
	Ⅱb	5	1			
	Ⅲa	10	4			
	Ⅲb	27	16			
	Ⅲc	6	5			
	Ⅳ	4	6			
Dose (%)	Oxaliplatin	89.55 ± 7.59	88.10 ± 8.50	Z = -0.7638	0.4449	a)
	Capecitabine	93.49 ± 9.41	92.40 ± 11.08	Z = -0.4466	0.6551	a)

Thrombocytopenia

The results of the comparison of pre-treatment factors associated with thrombocytopenia are shown in Table 2 and Table 7, and the results of the multivariate logistic regression analysis are shown in Table 3D. In patients with thrombocytopenia, the lymphocyte count (×10²/μL) and SII at the start of the first cycle showed significant differences between the Grade ≥1 group and the Grade 0 group (lymphocyte: Z = -2.0767, p = 0.0378; SII: Z = -2.6120, p = 0.0090) (Table 2). The results of multivariate logistic regression analysis identified lymphocyte count (OR = 0.4810, p = 0.0326) and SII (OR = 0.9910, p = 0.0382) as independent factors associated with the occurrence of thrombocytopenia (Table 3D). In the ROC analysis, lymphocyte counts ≤ 12.41 × 10²/μL and SII ≤ 477.901 were identified as cutoff values, and the AUC ranged from 0.63 to 0.67 (Table 4C). Based on the above, lymphocyte count and SII were identified as pre‑treatment factors associated with an increased risk of thrombocytopenia. The results of the ROC analysis for each toxicity (cutoff values, AUC, sensitivity, and specificity) are shown in Table 4.

**Table 7:** Pre‑treatment factors associated with thrombocytopenia (Grade ≥1) Values marked with an asterisk (*) are statistically significant (p < 0.05). Statistical tests used: a) Wilcoxon two-sample test; b) Pearson's chi-square test.

Variable		Grade 0	Grade ≥ 1	Statistic (Z / χ²)	p-value	
		n = 82	n = 26			
Age (years)		66.98 ± 10.40	66.38 ± 9.76	Z = -0.4495	0.6530	a)
Sex	Male	73	24	χ² = 7.7125	0.7189	b)
	Female	9	2			
BMI (kg/m²)		22.32 ± 3.98	21.95 ± 3.62	Z = -0.3090	0.7573	a)
Body surface area (m²)		1.56 ± 0.18	1.59 ± 0.21	Z = 0.4670	0.6404	a)
General condition (PS）	0	73	24	χ² = 7.7125	0.6296	b)
	1	9	2			
Smoking history	0	32	11	χ² = 1.3772	0.8478	b)
	1-199	9	1			
	200-399	6	2			
	400-699	11	3			
	700-	24	9			
Drinking history	None	27	7	χ² = 1.4117	0.7027	b)
	Occasional	17	4			
	Weekly	12	6			
	Everyday	26	9			
Cancer site	Upper colon	12	0	χ² = 7.6996	0.2166	b)
	Lower colon	5	1			
	Sigmoid colon	15	9			
	Transverse colon	13	6			
	Jejunum	1	0			
	Rectum	27	9			
	Cecum	10	1			
Stage	Ⅱa	20	4	χ² = 2.8166	0.6707	b)
	Ⅱb	4	2			
	Ⅲa	12	2			
	Ⅲb	30	13			
	Ⅲc	8	3			
	Ⅳ	8	2			
Dose (%)	Oxaliplatin	88.96 ± 8.04	89.35 ± 7.63	Z = 0.1113	0.9113	a)
	Capecitabine	92.43 ± 9.42	95.27 ± 11.51	Z = 1.9617	0.0498*	a)

## Discussion

In this study, we conducted an exploratory analysis to examine how pre-treatment hematological, inflammatory, and immune markers are associated with the development of hematological toxicity in colorectal cancer patients receiving CapeOX therapy. The results suggested that certain pre-treatment factors may serve as predictors for leukopenia, anemia, and thrombocytopenia.

With regard to leukopenia, SII was identified as the sole independent pre-treatment factor. SII is a composite inflammatory marker calculated from platelet, neutrophil, and lymphocyte counts and is known to reflect tumor-associated inflammation and immune status [5]. However, because SII is calculated directly from platelet, neutrophil, and lymphocyte counts and the outcomes include leukopenia and thrombocytopenia, the observed association may partly reflect low baseline hematologic reserve or mathematical coupling with the outcome rather than an independent inflammatory mechanism. Correlation analyses showed strong associations between SII and its component blood counts, supporting the need for cautious mechanistic interpretation. Therefore, the role of SII in hematologic toxicity should be considered exploratory and may primarily reflect baseline hematologic status rather than a distinct inflammatory pathway. In addition, lower pretreatment SII may indicate reduced marrow proliferative capacity or subclinical hematologic vulnerability, making patients more susceptible to chemotherapy‑induced leukocyte depletion. This provides a biological rationale for the observed association between low SII and leukopenia. In the field of gastrointestinal cancers, including colorectal cancer, SII has been reported to be associated with prognosis and chemotherapy toxicity [8]; its identification as a predictor of leukopenia in this study is consistent with existing findings. Since the pre-treatment inflammatory state may influence susceptibility to bone marrow suppression, SII is considered useful for risk stratification prior to the start of treatment.

Regarding neutropenia, although SIRI and NLR showed an association in univariate analysis, they did not remain as independent factors in multivariate analysis. First, it has been reported that neutropenia is strongly influenced by drug exposure, and it has been suggested that individual differences in the pharmacokinetics and exposure levels of anticancer drugs may contribute to the manifestation of toxicity [9]. Furthermore, it has been shown that impaired renal function affects drug clearance and exacerbates bone marrow suppression [10]. In addition, it is known that genetic background (polymorphisms in drug-metabolizing enzymes and transporters) is involved in the development of neutropenia [10]. Based on these findings, neutropenia is likely a multifactorial toxicity that is difficult to predict using pre-treatment factors alone. The results of this study suggest that it is difficult to adequately predict neutropenia based solely on pre-treatment inflammatory and immune markers.

Age, red blood cell count, and Hb were identified as independent predictors of hemoglobin decline. First, it has been reported that older adults are more prone to developing anemia during chemotherapy due to reduced bone marrow reserve and the effects of nutritional status [11]. Furthermore, it has been suggested that age-related fragility of hematopoietic function may influence chemotherapy toxicity [12]. Second, it has been shown that patients with low pre-treatment red blood cell counts are more susceptible to bone marrow suppression caused by anticancer drugs [13]. Similarly, multiple studies have reported that a low pre-treatment Hb level is a significant risk factor for the development of anemia during chemotherapy [14,15]. The results of this study indicate that a tendency toward anemia prior to treatment is a significant risk factor for a decrease in hemoglobin during treatment, reaffirming the importance of hematological evaluation before the start of treatment.

However, because baseline hemoglobin lies close to the CTCAE Grade 1 threshold used to define first‑cycle anemia, the observed association may partly reflect baseline status or mathematical coupling with the outcome rather than treatment‑induced toxicity. Lower baseline Hb was strongly associated with meeting the study’s anemia definition, and this relationship should not be interpreted as an independent causal marker of CapeOX‑induced anemia without analyses accounting for baseline anemia and absolute Hb change. Therefore, the finding should be considered exploratory and requires validation using clinically meaningful toxicity thresholds.

With regard to thrombocytopenia, lymphocyte count and SII were identified as independent factors. First, it has been reported that a low lymphocyte count prior to treatment is associated with the development of hematologic toxicity during chemotherapy [16]. Furthermore, it has been suggested that lymphocytopenia reflects immunological vulnerability and may contribute to the exacerbation of chemotherapy-related toxicity [17]. In addition, it has been shown that patients with low pre-treatment lymphocyte counts are more prone to myelosuppression, including thrombocytopenia [18]. This study suggested that low pre-treatment SII levels are associated with thrombocytopenia and that the pre-treatment inflammatory and immune status may be involved in the development of thrombocytopenia. The results of this study indicate that thrombocytopenia is a toxicity susceptible to the influence of immune and inflammatory states, suggesting that assessment of the pre-treatment immune status may be useful.

This study has several statistical limitations. Given its exploratory nature, this study conducted univariate analyses on numerous pre-treatment factors and included those showing statistically significant differences in the multivariate logistic regression analysis. Therefore, the possibility of Type I errors (false positives) associated with multiple comparisons cannot be ruled out. Furthermore, the number of events for some hematologic toxicities was small, particularly leukopenia (11 events) and neutropenia (15 events), raising concerns about the stability and robustness of the multivariable logistic regression estimates. In addition, the AUC values obtained from the ROC analysis were sometimes moderate, suggesting that the predictive model has limitations in terms of accuracy.

In addition, some categorical comparisons involved sparse cell counts, for which ordinary chi‑square testing is not optimal. These univariate results were therefore interpreted only as exploratory screening, and the potential impact of sparse data on the robustness of the p‑values represents a limitation of this study. Because extensive univariate testing increases the risk of type I error, these findings should be interpreted as exploratory and hypothesis‑generating.

The cutoff values derived in this study were obtained and evaluated using the same small dataset without internal or external validation. This approach may lead to optimistic AUC estimates and data‑specific thresholds. Therefore, the cutoff values should be considered exploratory and require independent validation in larger cohorts before being applied for clinical risk stratification.

A single Day 21 measurement may not necessarily capture the hematologic nadir, and this limitation should be considered when interpreting the association between pretreatment laboratory values and hematologic toxicity. Baseline dose reductions may have influenced the occurrence of hematologic toxicity, and this factor should be considered when interpreting the association between pretreatment laboratory values and toxicity.

In addition, because hematologic toxicity was defined by the absolute CTCAE grade at the end of cycle 1, the present analysis does not distinguish between new‑onset and worsening abnormalities. This is particularly relevant for hemoglobin, where the baseline value is also used as a predictor. Future studies should define toxicity based on new‑onset or worsening from baseline and incorporate clinically meaningful thresholds.

Furthermore, this study is a single-center retrospective analysis, and patient characteristics, treatment strategies, and the use of supportive care may vary depending on the specific characteristics of the institution. Therefore, caution is required when interpreting the results for generalization to other institutions, and validation through prospective studies involving a more diverse patient population is needed.

However, as an exploratory analysis, this study presents clinically useful hypotheses suggesting that pre-treatment factors may contribute to the development of hematologic toxicity. These findings serve as an important starting point that should be validated in future prospective and multicenter studies, and are expected to lead to the development of more accurate predictive models.

In addition, the multivariable logistic regression models in this exploratory analysis were developed with a limited number of events relative to the number of candidate predictors, and several hematologic and inflammatory variables (e.g., white blood cell count, neutrophil count, lymphocyte count, platelet count, SII, SIRI, NLR, red blood cell count, and hemoglobin) are mathematically and biologically correlated. These factors raise concerns about overfitting and multicollinearity, and the resulting ORs and cutoff values should be interpreted very cautiously as data-driven, exploratory estimates rather than definitive clinical thresholds. Future studies should adopt more parsimonious prespecified models and consider penalized logistic regression or other shrinkage approaches to improve model stability and generalizability.

Based on the above, it was suggested that some of the hematologic toxicities associated with CapeOX therapy can be predicted based on pre-treatment hematologic markers and inflammatory and immune markers, and that this may contribute to risk stratification prior to treatment initiation and the consideration of individualized supportive care.

## Conclusions

In this exploratory single‑center cohort, lower baseline SII was associated with Cycle 1 leukopenia, while lower baseline lymphocyte count and SII were associated with thrombocytopenia. Baseline hemoglobin and red blood cell count were strongly associated with meeting the study’s anemia definition. Because the models were data‑driven, the outcomes were predominantly Grade ≥1, and no external validation was performed, these findings should be considered hypothesis‑generating rather than clinically actionable predictors. Prospective multicenter studies with prespecified, parsimonious models and external validation are needed before these markers can be used to guide supportive care or treatment modification.

## Appendices

**Table 8:** Reference ranges for blood test values and nutritional and inflammatory indices CRP: C-reactive protein; AST: aspartate aminotransferase; ALT: alanine aminotransferase; Hb: hemoglobin; eGFR: estimated glomerular filtration rate; Cr: creatinine; Ccr: creatinine clearance; PNI: prognostic nutrition index; SIRI: systemic inflammatory response index; NLR: neutrophil-to-lymphocyte ratio; PLR: platelet-to-lymphocyte ratio; LMR: lymphocyte-to-monocyte ratio; SII: systemic immune inflammatory index

Variable	Reference range (normal value)
		Red blood cell (10^6^/μL)	Male 420–570 / Female 380–500
				White blood cell (10^3^/μL)	35–90
Basophil (10^3^/μL)	0-2
		Eosinophil (10^3^/μL)	0-6
Neutrophil (10^3^/μL)	40-75		
		Lymphocyte (10^3^/μL)	20-50
Monocyte (10^3^/μL)	2-10		
		Platelet (10^4^/μL)	15-35
Serum albumin (g/dL)	3.8-5.0		
		CRP (mg/dL)	<0.3
AST (IU/L)	13-33		
		ALT (IU/L)	8-42
Hb (g/dL)	Male 13.0–17.0 / Female 11.5–15.0		
		eGFR (mL/min/1.73m^2^)	≥60
Cr (mg/dL)	Male 0.65–1.07 / Female 0.46–0.79		
		Ccr (mL/min)	80-130
PNI	≥45(Good)		
SIRI	1.0-1.5 (clinical cut-off)
NLR	1.0-3.0
PLR	100-200
LMR	3.0-5.0
SII	≤500 (increased by inflammation and tumors)

**Table 9:** Baseline patient information by adverse effect observed at the end of one treatment cycle Values marked with an asterisk (*) are statistically significant (p < 0.05). Statistical tests used: a) Wilcoxon two-sample test; b) Pearson's chi-square test. One case involved a synchronous jejunal lesion. Although the jejunum is not a colorectal site, the patient received chemotherapy according to colorectal cancer protocols; therefore, the case was retained in the colorectal cancer cohort.

Variable		Leukopenia				Neutropenia				Hemoglobin decrease				Thrombocytopenia				
		Grade 0	Grade ≥ 1	Statistic (Z / χ²)	p-value	Grade 0	Grade ≥ 1	Statistic (Z / χ²)	p-value	Grade 0	Grade ≥ 1	Statistic (Z / χ²)	p-value	Grade 0	Grade ≥ 1	Statistic (Z / χ²)	p-value	
		n = 97	n = 11			n = 93	n = 15			n = 71	n = 37			n = 82	n = 26			
Age (years)		66.68 ± 10.40	68.27 ± 8.58	Z = 0.3863	0.6992	67.46 ± 10.21	63.00 ± 9.65	Z = -1.8007	0.0717	64.54 ± 10.81	71.24 ± 7.25	Z = 3.2595	0.0011*	66.98 ± 10.40	66.38 ± 9.76	Z = -0.4495	0.6530	a)
Sex	Male	55	4	χ² = 1.7112	0.1991	52	7	χ² = 0.1500	0.5044	46	13	χ² = 7.5776	0.0033*	73	24	χ² = 7.7125	0.7189	b)
	Female	42	7			41	8			25	24			9	2			
BMI (kg/m²)		22.10 ± 3.72	22.64 ± 2.99	Z =0.4571	0.6476	22.05 ± 3.72	23.41 ± 4.3	Z = 1.9632	0.0496*	23.24 ± 3.75	20.31 ± 3.17	Z = -3.9748	<0.0001*	22.32 ± 3.98	21.95 ± 3.62	Z = -0.3090	0.7573	a)
Body surface area (m²)		1.57 ± 0.20	1.55 ± 0.14	Z = -0.5180	0.6044	1.57 ± 0.19	1.59 ± 0.21	Z = 0.7639	0.4449	1.62 ± 0.18	1.47 ± 0.17	Z = -4.0201	<0.0001*	1.56 ± 0.18	1.59 ± 0.21	Z = 0.4670	0.6404	a)
General condition（PS）	0	85	10	χ² = 1.1357	0.5965	85	12	χ² = 3.5447	0.1756	66	31	χ² = 22.8371	0.0663	73	24	χ² = 7.7125	0.6296	b)
	1	8	1			8	3			5	6			9	2			
Smoking history	0	38	5	χ² = 0.0032	0.5965	38	5	χ² = 5.0055	0.2366	23	20	χ² = 25.4196	0.0463*	32	11	χ² = 1.3772	0.8478	b)
	1-199	9	1			8	2			1	4			9	1			
	200-399	6	2			6	2			8	0			6	2			
	400-699	13	1			10	4			12	2			11	3			
	700-	31	2			31	2			22	11			24	9			
Drinking history	None	30	2	χ² = 5.8957	0.1912	31	3	χ² = 1.9445	0.5847	16	18	χ² = 8.6613	0.0330*	27	7	χ² =1.4117	0.7027	b)
	Occasional	21	0			18	3			15	6			17	4			
	Weekly	17	1			16	2			12	6			12	6			
	Everyday	29	6			28	7			28	7			26	9			
Cancer site	Upper colon	12	0	χ² = 8.1922	0.2790	12	0	χ² = 7.7881	0.2540	4	8	χ² = 9.4878	0.2133	12	0	χ² =7.6996	0.2166	b)
	Lower colon	5	0			5	1			4	2			5	1			
	Sigmoid colon	21	3			23	1			19	5			15	9			
	Transverse colon	19	0			17	2			12	7			13	6			
	Jejunum	1	0			1	0			0	1			1	0			
	Rectum	32	4			28	8			25	9			27	9			
	Cecum	8	3			8	3			7	4			10	1			
Stage	Ⅱa	23	1	χ² = 4.3770	0.9702	21	3	χ² = 10.1572	0.2946	19	5	χ² = 6.5384	0.1722	20	4	χ² = 2.8166	0.6707	b)
	Ⅱb	5	1			3	3			5	1			4	2			
	Ⅲa	13	1			12	2			10	4			12	2			
	Ⅲb	36	7			36	7			27	16			30	13			
	Ⅲc	10	1			11	0			6	5			8	3			
	Ⅳ	10	0			10	0			4	6			8	2			
Dose (%)	Oxaliplatin	89.25 ± 7.74	87.36 ± 9.54	Z = -0.6094	0.5422	89.10 ± 7.98	89.09 ± 7.75	Z = -0.0355	0.9717	89.55 ± 7.59	88.10 ± 8.50	Z = -0.7638	0.4449	88.96 ± 8.04	89.35 ± 7.63	Z = 0.1113	0.9113	a)
	Capecitabine	92.90 ± 9.82	94.97 ± 11.59	Z = 1.0462	0.2954	92.8 ± 9.47	95.23 ± 12.86	Z = 0.9239	0.3555	93.49 ± 9.41	92.40 ± 11.08	Z = -0.4466	0.6551	92.43 ± 9.42	95.27 ± 11.51	Z = 1.9617	0.0498*	a)
